# Conformational Plasticity of the Active Site Entrance in *E. coli* Aspartate Transcarbamoylase and Its Implication in Feedback Regulation

**DOI:** 10.3390/ijms21010320

**Published:** 2020-01-03

**Authors:** Zhen Lei, Nan Wang, Hongwei Tan, Jimin Zheng, Zongchao Jia

**Affiliations:** 1 College of Chemistry, Beijing Normal University, Beijing 100875, China; leizhen@mail.bnu.edu.cn (Z.L.); wangnan@xzhmu.edu.cn (N.W.); hongwei.tan@bnu.edu.cn (H.T.); 2 Jiangsu Key Laboratory of Brain Disease Bioinformation, Research Center for Biochemistry and Molecular Biology, Xuzhou Medical University, Xuzhou 221004, China; 3 Department of Biomedical and Molecular Sciences, Queen’s University, Kingston, ON K7L3N6, Canada

**Keywords:** aspartate transcarbamoylase, structural biology, enzymatic kinetics, molecular dynamics, feedback mechanism

## Abstract

Aspartate transcarbamoylase (ATCase) has been studied for decades and *Escherichia coli* ATCase is referred as a “textbook example” for both feedback regulation and cooperativity. However, several critical questions about the catalytic and regulatory mechanisms of *E. coli* ATCase remain unanswered, especially about its remote feedback regulation. Herein, we determined a structure of *E. coli* ATCase in which a key residue located (Arg167) at the entrance of the active site adopted an uncommon open conformation, representing the first wild-type apo-form *E. coli* ATCase holoenzyme that features this state. Based on the structure and our results of enzymatic characterization, as well as molecular dynamic simulations, we provide new insights into the feedback regulation of *E. coli* ATCase. We speculate that the binding of pyrimidines or purines would affect the hydrogen bond network at the interface of the catalytic and regulatory subunit, which would further influence the stability of the open conformation of Arg167 and the enzymatic activity of ATCase. Our results not only revealed the importance of the previously unappreciated open conformation of Arg167 in the active site, but also helped to provide rationalization for the mechanism of the remote feedback regulation of ATCase.

## 1. Introduction

Aspartate transcarbamoylase (E.C.2.1.3.2, aspartate carbamoyltransferase, ATCase) catalyzes the second step of the de novo biosynthesis of pyrimidines in most of organisms, which is the carbamoylation reaction between carbamoyl phosphate (CP) and L-aspartate (Asp), generating N-carbamoyl-L-aspartate (CA) and a single phosphate group (Pi) [1,2,3]. The catalytic mechanism of ATCase is ordered: CP binds before aspartate and carbamoyl aspartate leaves before phosphate [4,5]. The pyrimidine biosynthesis pathway of *E. coli* ATCase is physiologically controlled by altering the catalytic activity of ATCase through both cooperativity and feedback regulation [6,7], which has been referred to as a “textbook example” for decades.

*E. coli* ATCase holoenzyme is a dodecamer composed of two catalytic trimers and three regulatory dimers. Under physiological conditions, *E. coli* ATCase can adopt two states at quaternary level: a low activity and affinity T (tense) state and a high activity and affinity R (relax) state, where an equilibrium exists between the T and R state [8,9,10]. *E. coli* ATCase exists mainly in the T state when no substrate binding occurs and CP binding causes only minor tertiary change of the catalytic subunit of ATCase. A low concentration of Asp cannot transform ATCase from the T to R state either. However, when the concentration of Asp increases to a certain level, most ATCase will be transformed to the R state to significantly enhance the catalyzing capacity of the carbamoylation reaction between CP and Asp, which is the mechanism of cooperativity [11,12]. The catalytic chain of ATCase can be divided into two domains: the CP domain and the Asp domain, and it is the closure of the two domains that trigger the T to R state transition of ATCase [5,13]. There are three important residues—Glu50, Arg167, and Arg234—in the active site that stabilize the domain closure by forming a salt bridge [14,15]. Arg167 also interacts with Asp and the intermediate product [16].

The feedback regulation of ATCase helps *E. coli* balance the levels of pyrimidines and purines in cells. CTP and UTP, the end products of de novo pyrimidine synthesis pathway, inhibit the activity of ATCase, whereas ATP and GTP promote it [17]. It has been found that the binding of pyrimidines or purines not only influences V_max_, but also causes a pronounced change of K_m_. In other words, pyrimidines or purines change the difficulty level for ATCase to transit from T to R state. Nevertheless, it is yet to be elucidated how pyrimidines and purines exert their effects because they bind at a position far away from the active site (~60 Å in *E. coli* ATCase). Furthermore, ATCase structures bound with pyrimidines or purines do not show obvious differences [18]. Thus, we hypothesized that there must be a yet-unknown mechanism that enables this feedback regulation.

In this work, we solved a structure of *E. coli* ATCase holoenzyme and observed that Arg167 adopts an uncommon open conformation. Upon analyzing all *E. coli* ATCase holoenzymes, we found only four other structures that adopt this conformation, in which two of them are due to mutations that destabilize the interaction between catalytic and regulatory subunit and the other two are due to the unusual binding of substrates/substrate analogs in the active site of ATCase. Therefore, the structure we solved here was the first wild-type apo-form *E. coli* ATCase holoenzyme that features this conformation. We further performed enzymatic assays, CASTp analysis, and molecular dynamic simulations to study this Arg167 open conformation and found it plays a key role in the catalytic and/or regulatory process of ATCase. Based on these results and the previous literature, we inferred a close relationship between this conformation and the feedback mechanism of *E. coli* ATCase. Taken together, we demonstrated the importance of the open conformation of Arg167 and provide insights into the feedback regulation of *E. coli* ATCase.

## 2. Results

### 2.1. The Structure of ATCase Holoenzyme with an Uncommon Open Conformation of Arg167 in Its Catalytic Subunit

**Figure 1:**
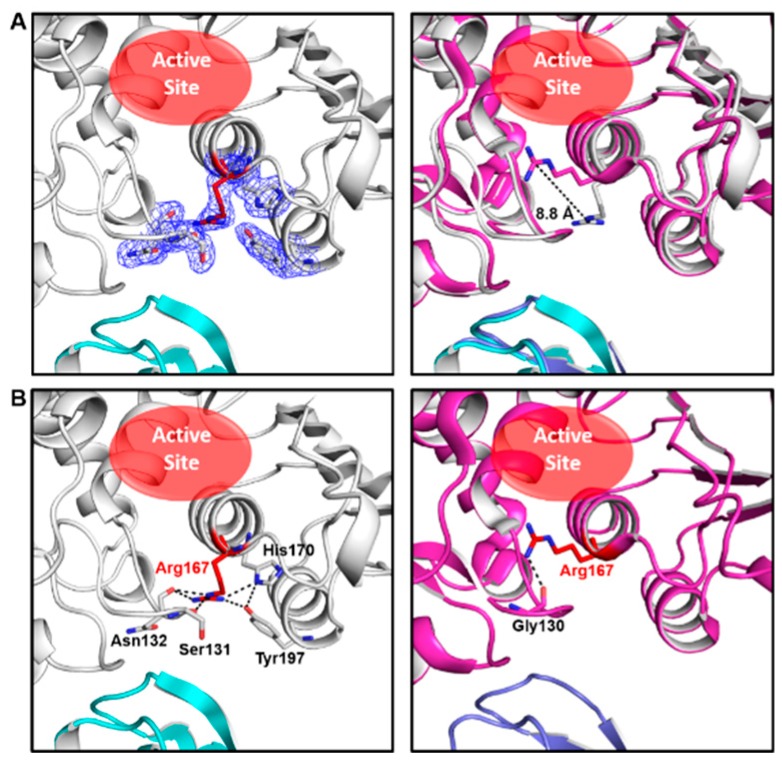
Our ATCase structure and its comparison with a previously solved ATCase structure adopting common conformation of Arg167. (**A**) Our structure of wild-type *E. coli* ATCase holoenzyme (left) and its alignment with an ATCase structure (PDB ID 1ZA1) with a closed conformation of Arg167 (right). In the left figure, the open conformation of Arg167 and its interacting residues are shown as sticks in electron density map (contoured at 1.0 σ), and Arg167 is colored in red, catalytic subunit in white, regulatory subunit in cyan. The active site is indicated by a red ellipse. In the right figure, the distance between different conformations of Arg167 is indicated and the position of the active site is also shown. (**B**) The interactions of open (left) and closed (right) conformation of Arg167. Arg167 and its interacting residues are labeled and shown as sticks and Arg167 is colored in red.

The crystal of ATCase belongs to the R32 space group and there is only one catalytic chain and one regulatory chain in an asymmetric unit. The statistics of X-ray data and refinement are listed in Table 1. In comparison to other ATCase holoenzyme structures, Arg167 in the catalytic chain of this structure adopts an uncommon open conformation, which shows an 8.8 Å conformational variation from the common closed conformation of Arg167 (Figure 1A). As a result of conformational plasticity, the side chain of Arg167 flips out to interact with the side chain of H170/Y197 and the main chain of Ser131/Asn132 in the open conformation, while the closed conformation of Arg167 only interacts with the main chain of Gly130 (Figure 1B). Among a total of ~60 ATCase holoenzyme structures, only four other ATCases adopt this conformation (PDB ID: 9ATC, 4E2F, 1R0C and 2AIR), in which two of them (PDB ID: 9ATC and 4E2F) have mutations destabilizing the T state and the other two (PDB ID: 1R0C and 2AIR) have substrate/substrate analog binding in the active site in an unusual way. Different from all the four ATCase holoenzyme structures referred to above, the structure we solved here contains neither mutation nor substrate/substrate analog. Therefore, it is the first wild-type apo-form ATCase holoenzyme structure that adopts this open conformation of Arg167. These facts suggest that the Arg167 open conformation is not an artifact and may play a role in the catalytic and/or regulatory process of ATCase holoenzyme.

### 2.2. Enzymatic Activity Assay of ATCase with Mutations of His170 and/or Tyr197

**Figure 2:**
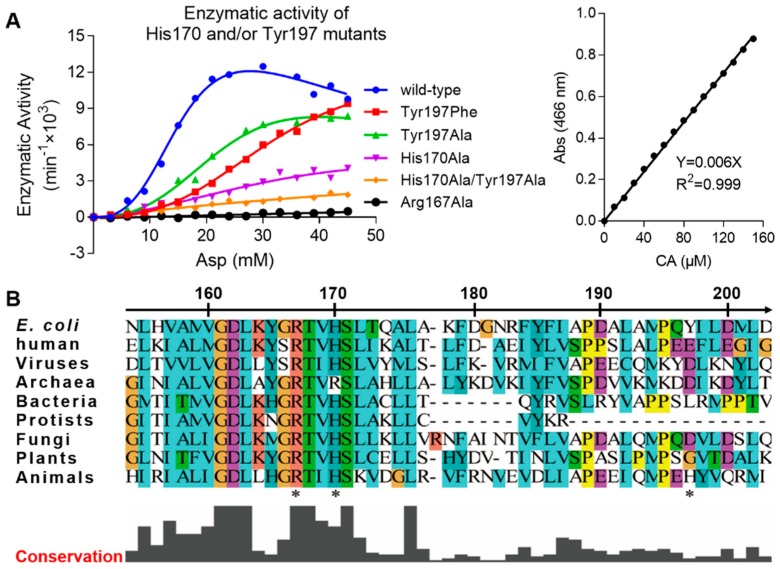
Enzyme kinetics of His170/Tyr197 mutants of *E. coli* ATCase holoenzyme and local sequence alignment of ATCase catalytic chain among different species. (**A**) The enzymatic kinetics curves of different His170/Tyr197 mutants of *E. coli* ATCase holoenzyme (left) and the standard curve used in this assay to convert absorption value into reaction velocity (right). (**B**) Local sequence alignment of ATCase catalytic chain containing Arg167, His170, and Tyr197 (indicated by stars below sequences) among different species from viruses to animals. In addition to *E. coli* and humans, the species chosen for each organism are listed as follows: viruses (*Only Syngen Nebraska virus 5*), bacteria (*Pseudothermotoga thermarum*, *DSM 5069*), Archaea (*Archaeoglobus veneficus*, *SNP6*), protists (*Trypanosoma grayi*), fungi (*Pyrenophora tritici-repentis*, *Pt-1C-BFP*), plants (*Solanum lycopersicum*), and animals (*Necator americanus*).

To study the importance of the Arg167 open conformation, we created several mutants and performed an enzymatic activity assay. The mutants were Arg167Ala, His170Ala, Tyr197Phe, Tyr197Ala, and His170Ala&Tyr197Ala. In this assay, wild-type and Arg167Ala ATCase were used as positive and negative controls, respectively. Enzymatic kinetic curves and the standard curve are shown Figure 2A. Corresponding parameters derived from these curves are listed in Table 2. The activity of all ATCase mutants was reduced, albeit to different degrees. The activity of the Tyr197 mutant decreased the least, the His170 mutant decreased more, and the double mutation of Tyr197 and His170 decreased the most. This result demonstrates that both His170 and Tyr197 residues are important and function cooperatively. Of these two residues, His170 is more important than Tyr197, which is not only evident from the enzymatic kinetics but also the sequence conservation in ATCases from different species (Figure 2B). Additionally, due to the fact that His170 and Tyr197 only interact with the open conformation of Arg167, our results reveal the importance of this open conformation of Arg167, which may play a role in the catalytic and/or regulatory process of ATCase.

### 2.3. Molecular Dynamics Simulation for the Stability of the Open and Closed Conformation of Arg167

**Figure 3:**
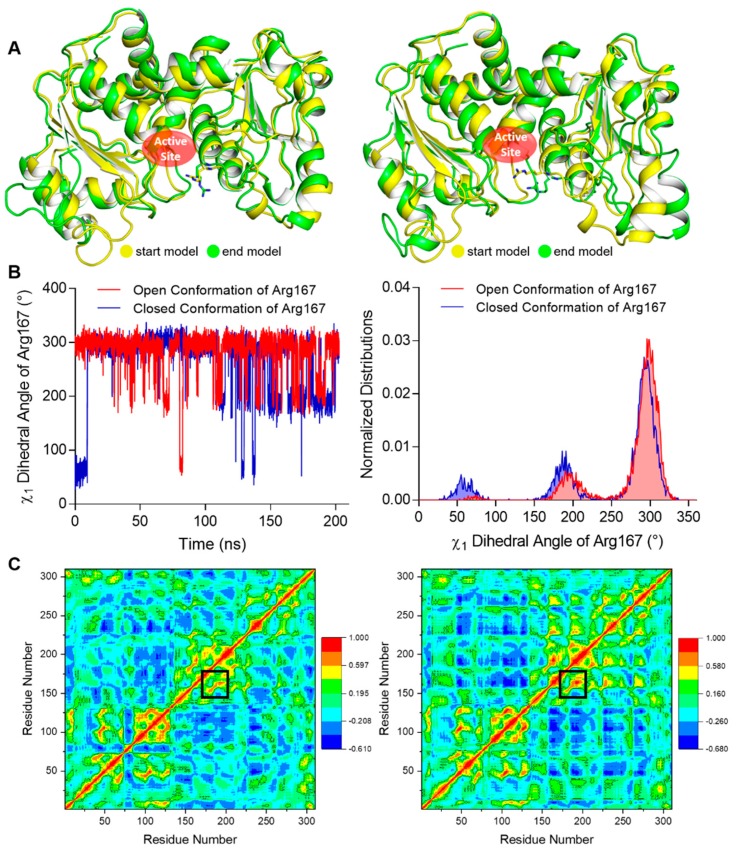
MD simulation results of our open Arg167 ATCase and the closed Arg167 ATCase. The results of the other two parallel groups of simulations are shown in Figures S1 and S2. (**A**) The structural alignment of the start and end conformations of a 200 ns MD simulation for ATCase with open (left) and closed (right) conformations of Arg167. In each alignment, start and end conformations are colored in yellow and green, respectively, and Arg167 is shown as sticks. (**B**) The comparison of χ_1_ dihedral angle fluctuation (left) and distribution (right) of Arg167 starting with open and closed conformations during the simulation. (**C**) The dynamic cross-correlation map in the MD simulation for ATCase with open (left) and closed (right) conformation of Arg167. In each map, the Cα correlation between Arg167 and His170/Tyr197 is indicated by black boxes.

To investigate the relative stability of the open and closed conformation of Arg167, we performed MD (Molecular Dynamics) simulations using our structure as well as an ATCase structure with the closed Arg167 conformation (PDB ID 1ZA1) as initial conformations for comparison. One catalytic chain was used in these simulations. The start and end conformations after a 200 ns simulation using open and closed conformations of Arg167 were aligned respectively (Figure 3A), which clearly shows that the open conformation maintained its conformation while the closed conformation changed to the open conformation after the simulation. We calculated the χ_1_ dihedral angles of Arg167 (the dihedral angle defined by N-CA-CB-CG of Arg) throughout the simulation, which can be used as an approximate indicator for the open (χ_1_ ≈ 295°) and closed (χ_1_ ≈ 64°) conformations of Arg167. The comparison of fluctuation and distribution of Arg167 χ_1_ dihedral angles are shown in Figure 3B, in which it is evident that the χ_1_ dihedral angle had sampled all possible χ_1_ values of the Arg side chain [19] in both cases, implying that the simulation experienced adequate sampling in finding the final side chain conformation. The χ_1_ dihedral angle fluctuation of Arg167 (Figure 3B, left) shows that the open conformation of Arg167 largely maintained its conformation throughout the simulation, while the closed conformation of Arg167 changed to open conformation after ~10 ns simulation and largely retained this state afterwards. This result is consistent with that shown in Figure 3A. The χ_1_ dihedral angle distribution of Arg167 (Figure 3B, right) clearly shows that the open conformation of Arg167 is the more stable one because it is the dominant conformation during the simulation as indicated by the highest peak in each case. To ensure the veracity of this result, the other two parallel groups of simulations with different initial velocities were performed and their results were consistent with this one, including the following DCCM (Dynamic Cross Correlation Map) analysis (Figures S1 and S2). This result indicates that the Arg167 open conformation is more stable than the closed conformation, which means ATCase probably adopts an open conformation of Arg167 before substrate entry.

**Figure 4:**
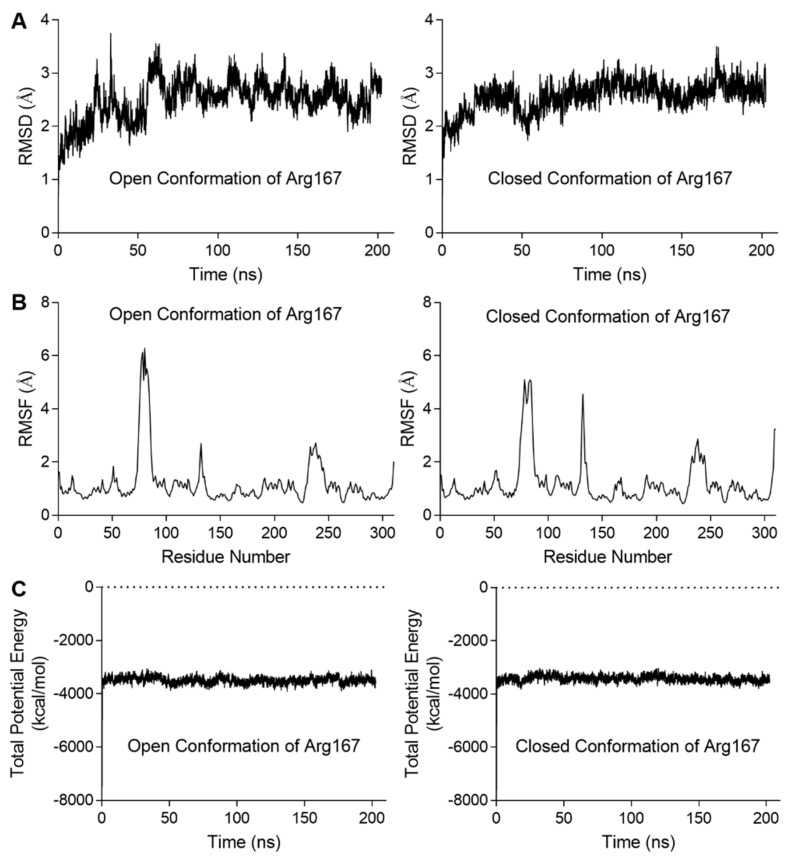
Diagrams depicting the MD simulation for the stability of ATCase with the open or closed conformations of Arg167. The results of the other two parallel groups of simulations are shown in Figures S3 and S4. (**A**) The rmsd variation of Cα during the simulation. In each case, the variation in rmsd was relatively steady at the end of simulation, indicating an equilibrium was achieved. (**B**) The rmsf of Cα variation averaged by residue. In each case, residues in the three peaks were located on 80s, 130s, and 240s loop. (**C**) The total potential energy variation during the simulation. In each case, the total potential energy becomes steady rapidly after equilibration process begins.

We also analyzed the correlation between Arg167 and His170/Tyr197 using a correlation map and found they are correlated in both simulations (Figure 3C). Furthermore, the correlation between Arg167 and His170/Tyr197 in closed Arg167 conformation was stronger than the open conformation (indicated by black boxes in Figure 3C), revealing that these two residues may play an important role in triggering the transformation of Arg167 from closed to open conformation. This finding indicates a close relationship between Arg167 and His170/Tyr197, which was reflected in the enzymatic activity experiments (Figure 2A). In addition, the diagrams depicting the variation in rmsd (root mean square deviation), rmsf (root mean square fluctuation), and total potential energy during these simulations are shown in Figure 4, showing an equilibrium state was achieved for each simulation. Diagrams depicting the other two parallel groups of MD simulations are shown in Figures S3 and S4.

### 2.4. CASTp Analysis of the Pocket of ATCase with Various Conformations of Arg167

**Figure 5:**
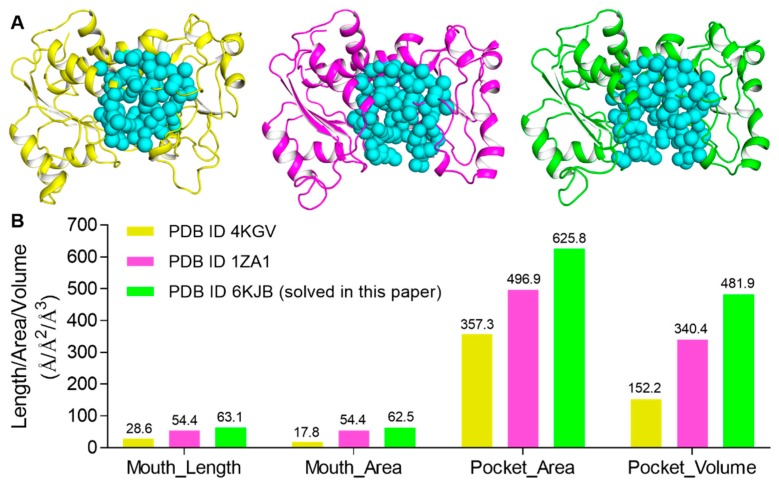
CASTp analysis of three Arg167 conformations in ATCase. (**A**) The ATCase structures of catalytic subunit used in CASTp analysis for the three conformations of Arg167 (from left to right: catalytic, closed, and open conformations of Arg167). (**B**) The mouth length/area and the pocket area/volume of ATCase of three conformations of Arg167, respectively. Values are labeled above each bar.

Besides the open and closed conformations of Arg167, there was also a third conformation of Arg167 (χ_1_ ≈ 44°), which is the catalytic conformation of Arg167. The third conformation of Arg167, together with Arg234, interacts with Glu50, forming a salt bridge to stabilize the domain closure of ATCase and catalyzes the reaction between CP and Asp. By using CASTp, we calculated the area/volume of the pocket of the active site and the length/area of the “mouth”, or entrance area, of ATCase for the three Arg167 conformations. The results are shown in Figure 5, in which the three ATCase structures of the catalytic subunit used in the calculation are shown in Figure 5A and the corresponding mouth/pocket values are shown and labeled in Figure 5B. ATCase with the catalytic conformation of Arg167 possessed the smallest mouth/pocket and ATCase with the open conformation of Arg167 possessed the largest one. This result suggests that the open conformation of Arg167 represents the most accessible state for substrate entrance. In consideration of the results we obtained in the enzymatic activity assay and MD simulation, we considered that ATCase adopts the open conformation of Arg167 before substrate entrance and that the substrate needs to break the interaction network of the Arg167 open conformation before entering the active site of ATCase.

## 3. Discussion

In this study, we solved a wild-type apo-form *E. coli* ATCase holoenzyme with an open conformation of Arg167 and investigated its importance using enzymatic activity assay, MD simulation, and CASTp analysis. Enzymatic activity assay showed that mutant His170 and/or Tyr197 that interact with the open conformation of Arg167 decrease the activity of ATCase. MD simulation indicated that the open conformation is more stable than the closed conformation. In addition, CASTp analysis revealed that ATCase with the open conformation of Arg167 possesses the largest mouth/pocket, which means this state is the most accessible conformation for substrate entry. Taking these results together, we believe that ATCase would adopt an Arg167 open conformation before substrate binding. To be more specific, the substrate here refers to the second one, Asp, because there is evidence demonstrating CP binding does not affect the conformation of Arg167 [5,20]. Asp needs to break the interactions of the open conformation of Arg167 and enter into the active site with the help of Arg167. This is the role that the Arg167 open conformation plays in the catalytic and/or regulatory process of ATCase.

**Figure 6:**
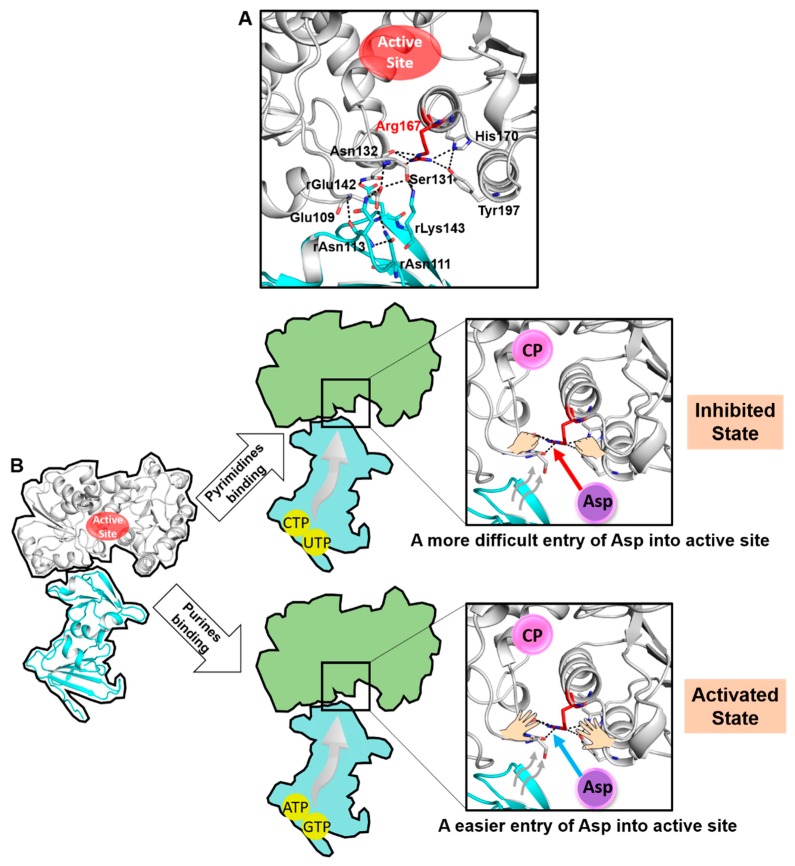
A proposed model of the feedback mechanism in *E. coli* ATCase. (**A**) Arg167 is involved in the large hydrogen-bonding network between the catalytic and regulatory subunit. (**B**) A model depicting the speculated feedback regulatory mechanism of ATCase.

In addition to the interaction between the open conformation of Arg167 and His170/Tyr197, the open conformation of Arg167 also interacts with the main chain of Ser131 and Asn132, and the latter further interacts with the regulatory subunit by rGlu142 and rLys143 (the r before residue label indicates that this residue belongs to the regulatory subunit). Previous literature indicates that the mutations of residues in the regulatory subunit (rAsn111, rAsn113, rGlu142 and rLys143) at the interface would either decrease/abolish the feedback regulation of purine/pyrimidine in *E. coli* ATCase [21] or destabilize the T state of *E. coli* ATCase [22]. Therefore, Arg167 is involved in the hydrogen bonding network at the interface between the catalytic and regulatory subunit (Figure 6A). We thus inferred a close relationship between the open conformation of Arg167 and the feedback regulation of ATCase.

Based on the key role of the Arg167 open conformation in the catalytic and/or regulatory process of ATCase and the analysis of its relationship with the feedback regulation, we proposed that the open conformation of Arg167 plays a significant role in the feedback regulatory process. A model depicting this mechanism is shown in Figure 6B. In general, we believe ATCase adopts the Arg167 open conformation before Asp entry. Asp needs to break the interaction network of Arg167 to enter and bind to the active site. The binding of pyrimidines or purines would affect the hydrogen-bonding network at the interface between the catalytic and regulatory subunit, strengthening or weakening the stability of the open conformation of Arg167, and thus makes it more difficult or easier for Asp to enter into the active site. This would, in turn, make the T to R transition more difficult or easier, thus enabling the feedback regulation of ATCase.

In conclusion, the structure we solved here, with an unusual open conformation of Arg167, helped us uncover its important functional implications in the catalytic and/or regulatory process of ATCase, which demonstrated that the largely neglected open conformation of Arg167 is not a dispensable state and further helped reveal a new mechanism to explain the poorly understood remote feedback regulation of *E. coli* ATCase.

## 4. Materials and Methods

### 4.1. Extractions of ATCase from E. coli

The extraction of wild-type *E. coli* ATCase holoenzyme begun with the culturing of BL21(DE3) strain at 37 °C. Bacteria pellet was collected by centrifuge and the medium was discarded. The pellet was resuspended by Buffer A (20 mM Tris-HCl pH 8.0, 150 mM NaCl, and 10% Glycerol) and lysed by sonication. The lysate was centrifuged with 14,000× *g* for 30 min and the precipitate was removed. For reasons that are unclear, the native form of ATCase lacking the His tag was still able to bind to nickel resin, although not strongly. The supernatant was thus added to the 2 mL Ni-NTA resin (Qiagen), followed by a slight wash with 10 mL Buffer A and eluted with 10 mL Buffer A supplied with 500 mM imidazole. Buffer exchange with Buffer A was performed by ultracentrifuge to discard imidazole. The mixture was concentrated to 1 mL and purified by HiLoad Superdex 200 (GE) with Buffer A. Protein in peak fractions was collected and concentrated for crystallization screening.

### 4.2. Crystallization and Structure Determination

The preliminary crystallization conditions for native ATCase were screened by the sparse matrix method [23] and hanging drop vapor diffusion method was then used to improve the quality of the preliminary crystal hits. Hanging drops were set up with 2 μL of protein solution mixed with 2 μL of well solution. The primary optimal crystallization condition in the reservoir was 0.1 M MES, pH 6.0, 10% glycerol, and 10% PEG 8000. Crystals appeared in 2 days and grew to full size within 10 days. After that, crystals were flash frozen in liquid nitrogen for X-ray diffraction data collection.

The diffraction datasets for ATCase crystals were collected at BL17U1 beamline of Shanghai Synchrotron Radiation Facility [24] at 0.979 Å. Datasets were processed by HKL2000 [25]. Molecular replacement was performed by Phaser program [26] using a previous T state *E. coli* ATCase structure (PDB ID 1ZA1) as a search template. Subsequent refinement was carried out by REFMAC5 within CCP4 suite of programs [27] and Phenix [28], as well as Coot [29] for manual corrections.

### 4.3. Recombinant Expressions and Purifications of E. coli ATCase and Mutants

Wild-type ATCase cDNA of the catalytic and regulatory chain was obtained by PCR (Qiagen Kit) using BL21(DE3) strain genome as a template. After that, catalytic chain cDNA and regulatory chain cDNA were ligated to pET28b and pET22b vectors, respectively. In consideration of the weak binding of native ATCase with nickel resin, a 6× His tag was retained on catalytic chain plasmid. *E. coli* ATCase holoenzyme transformant was obtained by co-transforming the plasmids of the catalytic chain and regulatory chain into BL21(DE3) together, which was then cultured in 2 L LB (Lysogeny Broth) medium and induced by 1 mM IPTG (Isopropyl β-d-1-thiogalactopyranoside) when OD_600_ = 1.0. The bacteria pellet was then collected by centrifuging and the medium was discarded. The pellet was resuspended by Buffer A and lysed by sonication. The lysate was centrifuged with 14,000 × *g* for 30 min and the supernatant was added to the 2 mL Ni-NTA resin (Qiagen). After washing with 50mL Buffer A and 50 mL Buffer A supplied with 30 mM imidazole, protein was eluted with 10 mL Buffer A supplied with 300 mM imidazole. Protein in each step was monitored by SDS-PAGE and the protein concentration was measured by NonoPhotometer P-Class (IMPLEN) with a molar extinction coefficient of 0.59 cm^2^/mg. All *E. coli* ATCase mutants were obtained using a site-directed mutation kit (Qiangen) and were expressed/purified the same as above.

### 4.4. Activity Assay of ATCase and Mutants

An activity assay was performed colorimetrically, as previously reported [30]. Briefly, L-aspartate (AMRESCO) and carbamoyl phosphate (Sigma) were all dissolved in Buffer B (50mM Tris-Acetate pH 8.3). A final concentration of 0–45 mM L-aspartate and 6 nM *E. coli* ATCase or mutants were added into a 2 mL Eppendorf tube and Buffer B was added to obtain the final volume of 450 μL. These tubes were equilibrated to 25 °C in a water bath for 10 min. Assays were initiated by the addition of 50 μL 48 mM CP into the tube and allowed to proceed for 6 min. After that, the reaction was terminated by adding a termination agent (also the color agent), which contains antipyrine solution (5 g/L in 50% sulfuric acid) and 2,3-butadionemonoxime solution (8 g/L in 5% acetic acid) in a 2:1 ratio. The mixture was protected from light at room temperature for 16 h and incubated in a 45 °C water bath for 60 min. The final readout was determined by measuring the absorbance at 466 nm and transformed into reaction velocity according to a standard curve using the same approach with *N*-Carbamoyl-dl-Aspartate (TCI) as the standard product. Datasets were fitted with the Hill equation with/without substrate inhibition modification, as previously reported [31], according to different situations. V_max_, K_m_, and n_H_, and corresponding standard errors were calculated from these Hill equations and graphs were plotted by GraphPad Prism 7.00.

### 4.5. Molecular Dynamic Simulation and CASTp Analysis

MD simulations were performed using the Amber 16/AmberTools16 package [32], including three steps: pre-process, simulation, and post-process. For pre-processing, tleap was used to generate the topology and coordinate files for each system. During this process, ff14SB force field parameters [33] were used for protein atoms and TIP3P parameters [34] were used for water molecules. Each system was neutralized by Na^+^ or Cl^−^ ions and was explicitly solvated by using the TIP3P water potential inside a box of water molecules with a minimum solute-wall distance of 10 Å. For MD simulation, the CUDA version of PMEMD (particle mesh Ewald molecular dynamics) [35] was used on GPUs and the protocol is described as follows. First, the entire system was energy minimized to remove unfavorable contacts by six rounds of minimizations in the NVT ensemble, in which the protein atoms were restricted by Cartesian restraints decreasing from 0.1 to 0 kcal/(mol·Å^2^). In each round of minimization, the maximum number of cycles was set to 10,000, and the method of minimization was changed from steepest descent to conjugate gradient after 5000 cycles. Long-range electrostatic interactions were computed using the particle mesh Ewald (PME) method and non-bonded interactions were cut off at 10 Å (same for all following steps). Second, the energy-minimized structure was heated using the Langevin thermostat from 0 to 310 K in the NVT ensemble over 200 ps with a thermostat coupling constant of 0.5 ps. Periodic boundary conditions and wrapping were used during simulation with a time step of 2 fs (same for all following steps). Third, an unrestrained equilibration of 2 ns was carried out in the NPT ensemble, in which the temperature and pressure were allowed to fluctuate around 310 K and 1 bar, using Langevin thermostat and Berendsen barostat. Finally, an unrestrained production run of 200 ns was carried out, using the same conditions as the equilibration step, and trajectory snapshots were saved every 40 ps. For post-processing, CPPTRAJ was used to analyze the trajectory files, including calculation of the rmsd, rmsf, and total energy during the simulations, as well as generation of the dynamic cross-correlation maps and dihedral distributions of Arg167. All diagrams were plotted by GraphPad Prism 7.00.

Additionally, computed atlas of surface topography of proteins (CASTp) program [36] was used to determine the area/volume of the binding pocket and the length/area of the pocket mouth. Probe radius used in these analyses was the default value (1.4 Å) and the solvent-accessible method was used in these CASTp analyses.

## Figures and Tables

**Table 1:** X-ray diffraction data and refinement statistics of *E. coli* ATCase holoenzyme *^a^*.

Item	Value
**Data collection statistics**	
Wavelength (Å)	0.979
Space group	R32
Resolution (Å)	30.77–2.06 (2.13–2.06) *^b^*
R_meas_	0.127 (0.865)
Average (I/σ)	15.8 (3.0)
Redundancy	6.8 (7.3)
Completeness (%)	98.8 (95.8)
Unit cell	
a, b, c (Å)	129.7, 129.7, 198.0
α, β, γ (°)	90, 90, 120
**Refinement statistics**	
Resolution (Å)	30.77–2.06
Reflections	39,504 (3808)
R_work_/R_free_	0.18/0.21
Mean B value (Å^2^)	47.3
Number of atoms	
Protein	3387
Zinc	1
Water	326
RMS (Root-Mean-Square) deviations	
Bond lengths (Å)	0.006
Angles (°)	1.07

*^a^* This structure has been deposited to the PDB (Protein Data Bank) database with accession number: 6KJB. *^b^* Values in parentheses correspond to the highest-resolution shell.

**Table 2:** V_max_, K_m_, and n_H_ of ATCase variants.

ATCase Type	V_max_ (min^−1^ × 10^3^)	K_m_ (mM)	n_H_
wild-type ATCase	13.09 ± 0.26	13.84 ± 0.21	4.19 ± 0.23
Tyr197Phe mutant	13.34 ± 1.02	32.98 ± 2.02	2.76 ± 0.18
Tyr197Ala mutant	8.80 ± 0.50	19.22 ± 0.92	3.67 ± 0.70
His170Ala mutant	7.11 ± 4.06	38.76 ± 26.89	1.64 ± 0.55
His170Ala&Tyr197Ala mutant	4.93 ± 4.44	66.37 ± 88.54	1.17 ± 0.35
Arg167Ala mutant *^a^*	-	-	-

*^a^* This group is the negative control which could not be fitted with the Hill equation and corresponding V_max_, K_m_ and n_H_ were not provided.

## Supplementary Materials

Supplementary materials can be found at https://www.mdpi.com/1422-0067/21/1/320/s1.

## References

[1] Lowenstein J.M., Cohen P.P. (1956). Studies on the biosynthesis of carbamylaspartic acid. J. Biol. Chem..

[2] Jones M.E., Spector L., Lipmann F. (1955). Carbamyl Phosphate, The Carbamyl Donor In Enzymatic Citrulline Synthesis1. J. Am. Chem. Soc..

[3] Reichard P., Hanshoff G. (1956). Aspartate Carbamyl Transferase from Escherichia-Coli. Acta Chem. Scand..

[4] Wedler F.C. (1974). Mechanisms of substrate binding with glutamine synthetase. Equilibrium isotope exchanges with the ovine brain, pea seed, and Escherichia coli enzymes. J. Biol. Chem..

[5] Wang J., Stieglitz K.A., Cardia J.P., Kantrowitz E.R. (2005). Structural basis for ordered substrate binding and cooperativity in aspartate transcarbamoylase. Proc. Natl. Acad. Sci. USA.

[6] Lipscomb W.N., Kantrowitz E.R. (2012). Structure and mechanisms of Escherichia coli aspartate transcarbamoylase. Acc. Chem. Res..

[7] Kantrowitz E.R. (2012). Allostery and cooperativity in Escherichia coli aspartate transcarbamoylase. Arch. Biochem. Biophys..

[8] Howlett G.J., Blackburn M.N., Compton J.G., Schachman H.K. (1977). Allosteric regulation of aspartate transcarbamoylase. Analysis of the structural and functional behavior in terms of a two-state model. Biochemistry.

[9] Gerhart J.C., Pardee A.B. (1962). The enzymology of control by feedback inhibition. J. Biol. Chem..

[10] Fetler L., Kantrowitz E.R., Vachette P. (2007). Direct observation in solution of a preexisting structural equilibrium for a mutant of the allosteric aspartate transcarbamoylase. Proc. Natl. Acad. Sci. USA.

[11] Gerhart J.C., Pardee A.B. (1964). Aspartate Transcarbamylase, an Enzyme Designed for Feedback Inhibition. Fed. Proc..

[12] Monod J., Wyman J., Changeux J.P. (1965). On the Nature of Allosteric Transitions: A Plausible Model. J. Mol. Biol..

[13] Ladjimi M.M., Kantrowitz E.R. (1988). A possible model for the concerted allosteric transition in Escherichia coli aspartate transcarbamylase as deduced from site-directed mutagenesis studies. Biochemistry.

[14] Stebbins J.W., Zhang Y., Kantrowitz E.R. (1990). Importance of residues Arg-167 and Gln-231 in both the allosteric and catalytic mechanisms of Escherichia coli aspartate transcarbamoylase. Biochemistry.

[15] Newton C.J., Kantrowitz E.R. (1990). Importance of domain closure for homotropic cooperativity in Escherichia coli aspartate transcarbamylase. Biochemistry.

[16] Ke H.M., Lipscomb W.N., Cho Y.J., Honzatko R.B. (1988). Complex of *N*-phosphonacetyl-l-aspartate with aspartate carbamoyltransferase. X-ray refinement, analysis of conformational changes and catalytic and allosteric mechanisms. J. Mol. Biol..

[17] Cockrell G.M., Zheng Y., Guo W., Peterson A.W., Truong J.K., Kantrowitz E.R. (2013). New paradigm for allosteric regulation of Escherichia coli aspartate transcarbamoylase. Biochemistry.

[18] Stevens R.C., Gouaux J.E., Lipscomb W.N. (1990). Structural consequences of effector binding to the T state of aspartate carbamoyltransferase: Crystal structures of the unligated and ATP- and CTP-complexed enzymes at 2.6-A resolution. Biochemistry.

[19] Lovell S.C., Word J.M., Richardson J.S., Richardson D.C. (2000). The penultimate rotamer library. Proteins.

[20] Ruiz-Ramos A., Velazquez-Campoy A., Grande-Garcia A., Moreno-Morcillo M., Ramon-Maiques S. (2016). Structure and Functional Characterization of Human Aspartate Transcarbamoylase, the Target of the Anti-tumoral Drug PALA. Structure.

[21] Eisenstein E., Markby D.W., Schachman H.K. (1989). Changes in stability and allosteric properties of aspartate transcarbamoylase resulting from amino acid substitutions in the zinc-binding domain of the regulatory chains. Proc. Natl. Acad. Sci. USA.

[22] Eisenstein E., Markby D.W., Schachman H.K. (1990). Heterotropic effectors promote a global conformational change in aspartate transcarbamoylase. Biochemistry.

[23] Jancarik J., Scott W.G., Milligan D.L., Koshland D.E., Kim S.H. (1991). Crystallization and preliminary X-ray diffraction study of the ligand-binding domain of the bacterial chemotaxis-mediating aspartate receptor of Salmonella typhimurium. J. Mol. Biol..

[24] Wang Q.-S., Zhang K.-H., Cui Y., Wang Z.-J., Pan Q.-Y., Liu K., Sun B., Zhou H., Li M.-J., Xu Q. (2018). Upgrade of macromolecular crystallography beamline BL17U1 at SSRF. Nucl. Sci. Tech..

[25] Otwinowski Z., Minor W. (1997). Processing of X-ray diffraction data collected in oscillation mode. Methods Enzymol..

[26] Storoni L.C., McCoy A.J., Read R.J. (2004). Likelihood-enhanced fast rotation functions. Acta Crystallogr. Sect. D Biol. Crystallogr..

[27] Vagin A.A., Steiner R.A., Lebedev A.A., Potterton L., McNicholas S., Long F., Murshudov G.N. (2004). REFMAC5 dictionary: Organization of prior chemical knowledge and guidelines for its use. Acta Crystallogr. Sect. D Biol. Crystallogr..

[28] Adams P.D., Afonine P.V., Bunkóczi G., Chen V.B., Davis I.W., Echols N., Headd J.J., Hung L.-W., Kapral G.J., Grosse-Kunstleve R.W. (2010). PHENIX: A comprehensive Python-based system for macromolecular structure solution. Acta Crystallogr. Sect. D Biol. Crystallogr..

[29] Emsley P., Cowtan K. (2004). Coot: Model-building tools for molecular graphics. Acta Crystallogr. Sect. D Biol. Crystallogr..

[30] Pastra-Landis S.C., Foote J., Kantrowitz E.R. (1981). An improved colorimetric assay for aspartate and ornithine transcarbamylases. Anal. Biochem..

[31] Pastra-Landis S.C., Evans D.R., Lipscomb W.N. (1978). The effect of pH on the cooperative behavior of aspartate transcarbamylase from Escherichia coli. J. Biol. Chem..

[32] Case D., Betz R., Cerutti D.S., Cheatham T., Darden T., Duke R., Giese T.J., Gohlke H., Götz A., Homeyer N. (2016). Amber 2016.

[33] Maier J.A., Martinez C., Kasavajhala K., Wickstrom L., Hauser K.E., Simmerling C. (2015). ff14SB: Improving the Accuracy of Protein Side Chain and Backbone Parameters from ff99SB. J. Chem. Theory Comput..

[34] Price D.J., Brooks C.L. (2004). A modified TIP3P water potential for simulation with Ewald summation. J. Chem. Phys..

[35] Salomon-Ferrer R., Götz A.W., Poole D., Le Grand S., Walker R.C. (2013). Routine Microsecond Molecular Dynamics Simulations with AMBER on GPUs. 2. Explicit Solvent Particle Mesh Ewald. J. Chem. Theory Comput..

[36] Tian W., Chen C., Lei X., Zhao J., Liang J. (2018). CASTp 3.0: Computed atlas of surface topography of proteins. Nucleic Acids Res..

