# Supplementary material for: Conformational Plasticity of the Active Site Entrance in E. coli Aspartate Transcarbamoylase and Its Implication in Feedback Regulation
Source: Int J Mol Sci. 2020 Jan 3;21(1):320. doi: 10.3390/ijms21010320 (PMC6981877; doi:10.3390/ijms21010320)
Supplement: Supplementary file 1 [file ijms-21-00320-s001.pdf]

Supplementary Material for

Conformational plasticity of the active site entrance in *E. coli* aspartate transcarbamoylase and its implication in feedback regulation

Zhen Lei [1], Nan Wang [1,2], Hongwei Tan [1], Jimin Zheng [1,*] and Zongchao Jia [3,*]

1 College of Chemistry, Beijing Normal University, Beijing, 100875, China
2 Jiangsu Key Laboratory of Brain Disease Bioinformation, Research Center for Biochemistry and Molecular Biology, Xuzhou Medical University, Xuzhou 221004, China
3 Department of Biomedical and Molecular Sciences, Queen's University, Kingston, ON K7L3N6, Canada
* Correspondence: jimin_z@bnu.edu.cn, Tel: +86-010-58806002 (J.Z.); jia@queensu.ca, Tel: +1-613-5336277 (Z.J.)

Results of the other two parallel groups of MD simulations

To ensure the veracity of the results of MD simulations, triplicates of MD simulations with different initial velocities were performed. Besides the one in the main text, the other two are shown herein. As indicated by the results of the MD simulation in the main text, the simulation from 100-200 ns does not provide further valuable information. Therefore, we performed analysis of 0-100 ns simulation for these two groups of MD simulations to verify the consistency among all the three independent groups. Results are shown below (Figures S1 to S4) and they are highly consistent with the results in the main text.

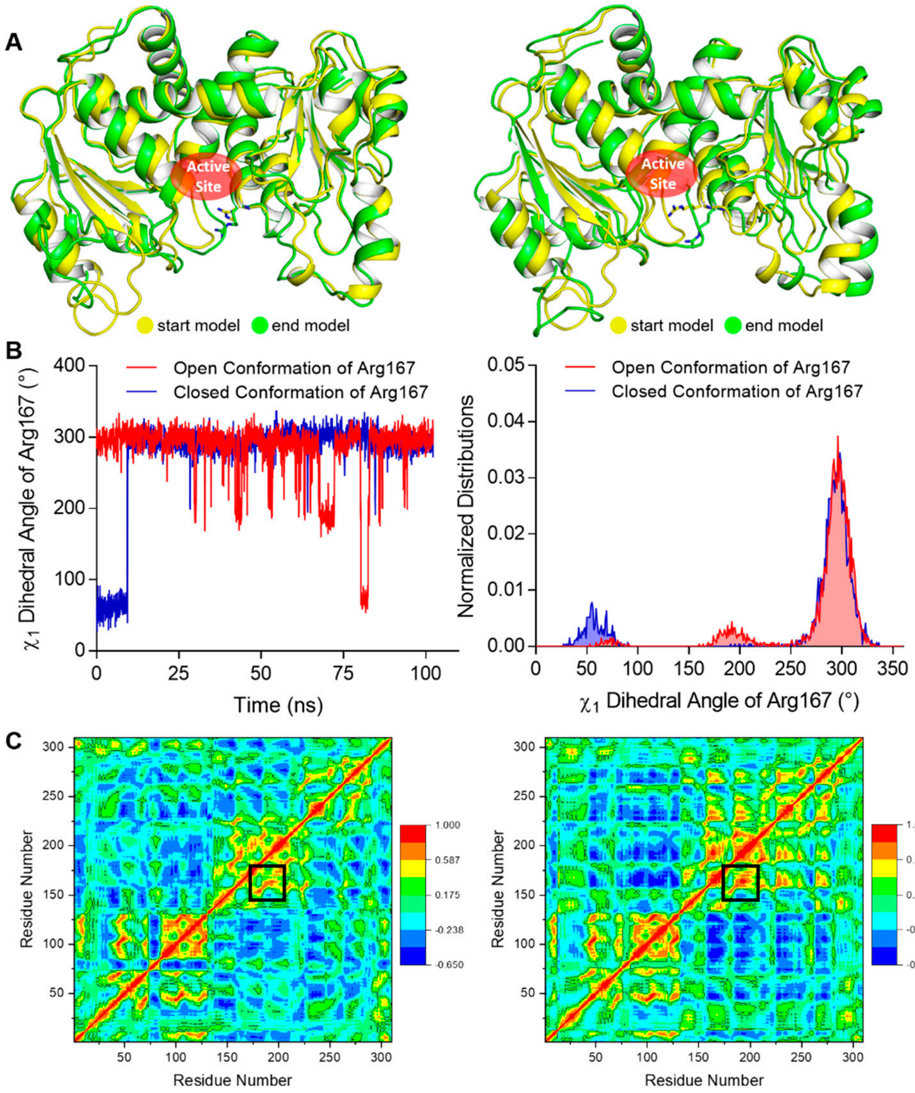

Figure S1. The MD simulation results of the second parallel group of our open Arg167 ATCase and the closed Arg167 ATCase, related to Figure 3. (**A**) The structural alignment of start and end conformations of the MD simulation for ATCase with open (left) and closed (right) conformation of Arg167. In each alignment, start and end conformations are colored in yellow and green respectively; Arg167 is shown as sticks. (**B**) The comparison of χ_1 dihedral angle fluctuation (left) and distribution (right) of Arg167 starting with open and closed conformation during the simulation. (**C**) The dynamic cross-correlation map in the MD simulation for ATCase with open (left) and closed (right) conformation of Arg167. In each map, the Cα correlation between Arg167 and His170/Tyr197 is indicated by black boxes.

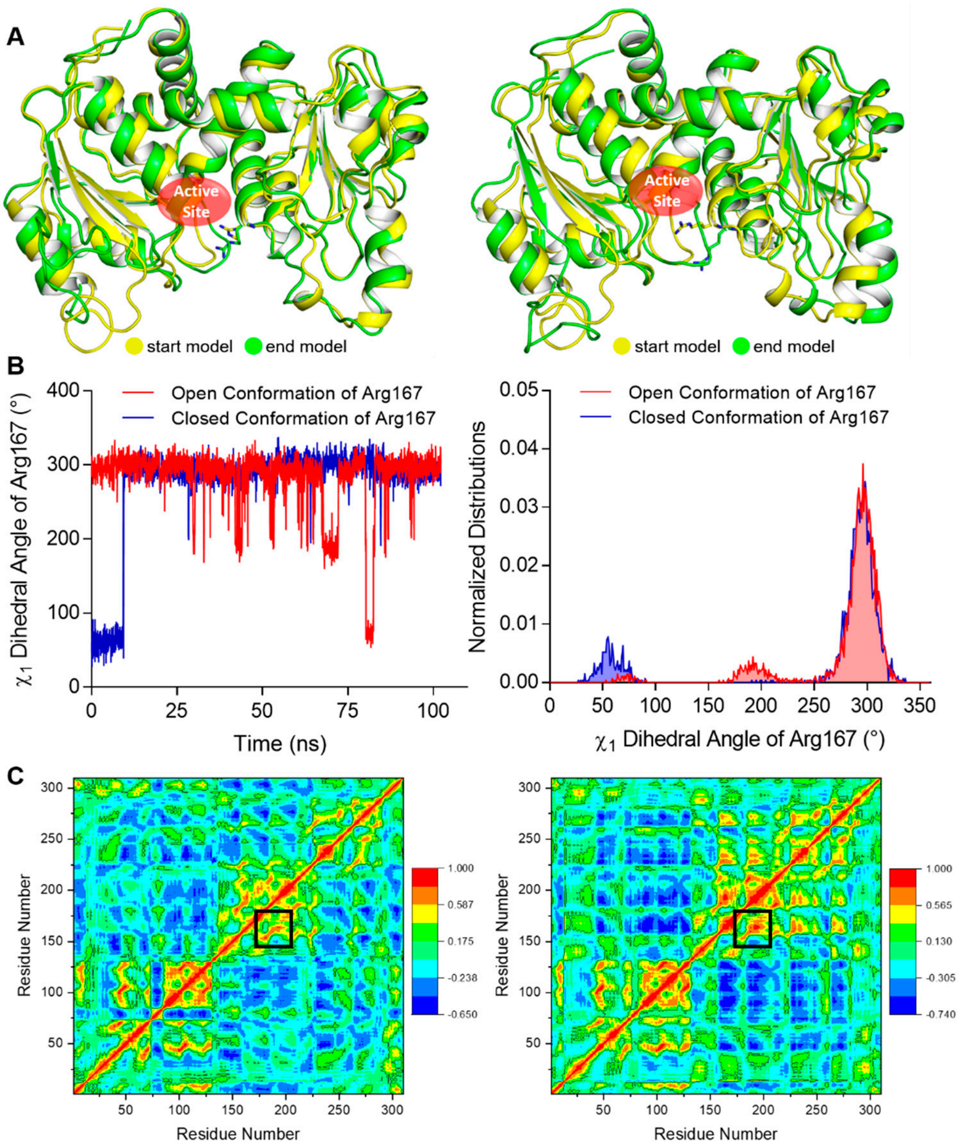

Figure S2. The MD simulation results of the third parallel group of our open Arg167 ATCase and the closed Arg167 ATCase, related to Figure 3. (**A**) The structural alignment of start and end conformations of the MD simulation for ATCase with open (left) and closed (right) conformation of Arg167. In each alignment, start and end conformations are colored in yellow and green respectively; Arg167 is shown as sticks. (**B**) The comparison of χ_1 dihedral angle fluctuation (left) and distribution (right) of Arg167 starting with open and closed conformation during the simulation. (**C**) The dynamic cross-correlation map in the MD simulation for ATCase with open (left) and closed (right) conformation of Arg167. In each map, the Cα correlation between Arg167 and His170/Tyr197 is indicated by black boxes.

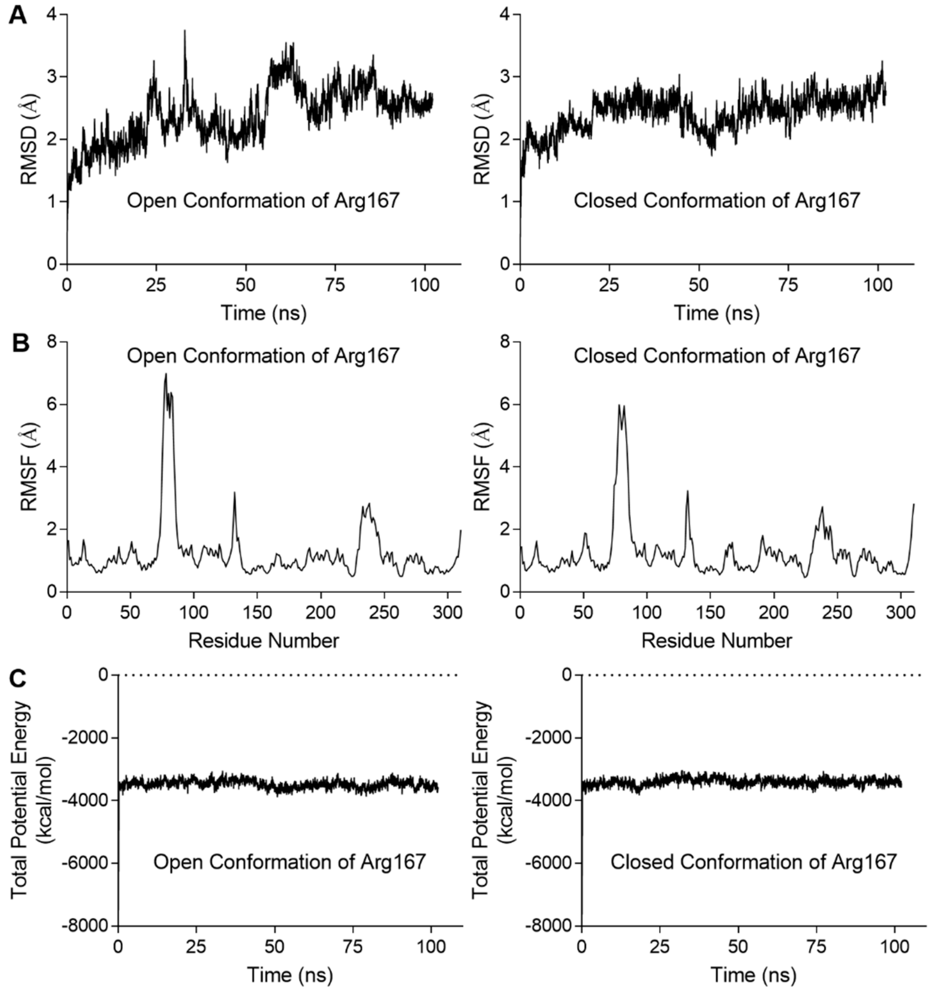

Figure S3. Diagrams depicting the second parallel group of MD simulations for the stability of ATCase with the open or closed conformation of Arg167, related to Figure 4. (**A**) The rmsd variation of Cα during the simulation. In each case, the variation of rmsd is relatively steady when comes to the end of simulation, indicating an equilibrium was achieved. (**B**) The rmsf of Cα variation averaged by residue. In each case, residues in the three peaks are located on 80's loop, 130's loop, and 240's loop. (**C**) The total potential energy variation during the simulation. In each case, the total potential energy becomes steady rapidly after equilibration process begins.

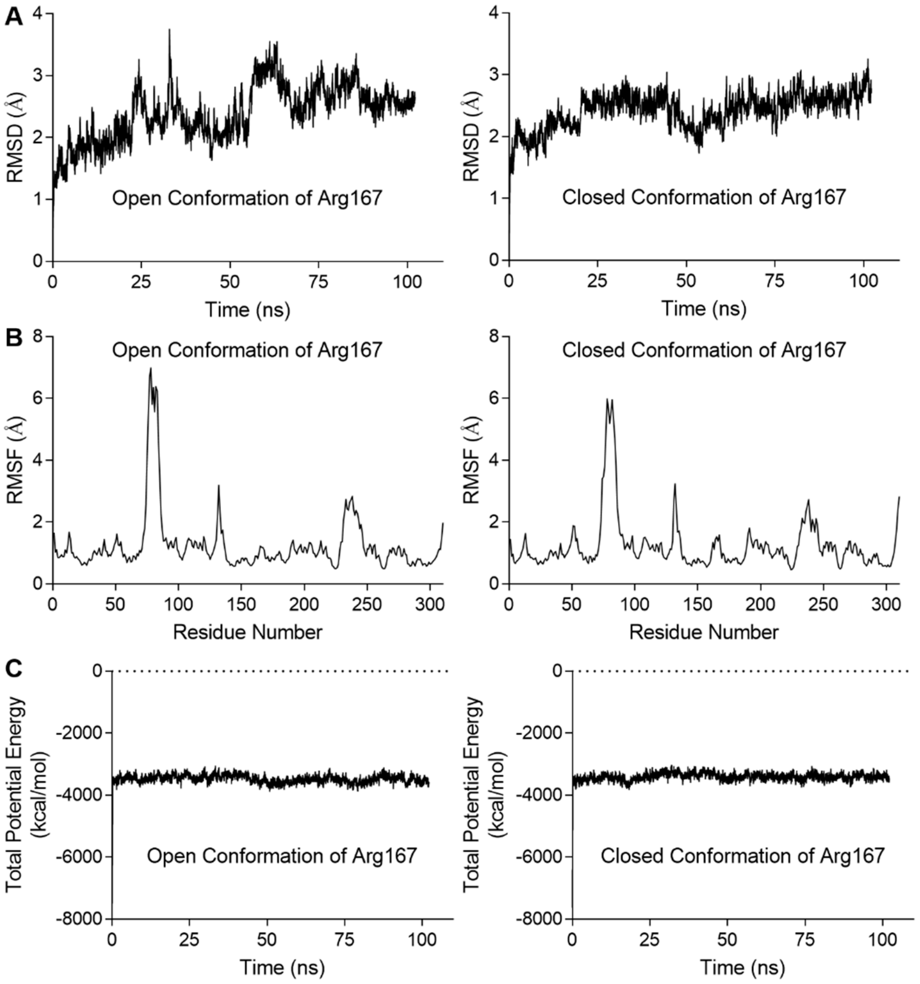

Figure S4. Diagrams depicting the third parallel group of MD simulations for the stability of ATCase with the open or closed conformation of Arg167, related to Figure 4. (**A**) The rmsd variation of Cα during the simulation. In each case, the variation of rmsd is relatively steady when comes to the end of simulation, indicating an equilibrium was achieved. (**B**) The rmsf of Cα variation averaged by residue. In each case, residues in the three peaks are located on 80's loop, 130's loop, and 240's loop. (**C**) The total potential energy variation during the simulation. In each case, the total potential energy becomes steady rapidly after equilibration process begins.
